# From Health Advice to Taboo: Community Perspectives on the Treatment of Sleeping Sickness in the Democratic Republic of Congo, a Qualitative Study

**DOI:** 10.1371/journal.pntd.0003686

**Published:** 2015-04-09

**Authors:** Alain Mpanya, David Hendrickx, Sylvain Baloji, Crispin Lumbala, Raquel Inocêncio da Luz, Marleen Boelaert, Pascal Lutumba

**Affiliations:** 1 Programme National de Lutte contre la Trypanosomiase Humaine Africaine, Kinshasa, Democratic Republic of Congo; 2 Institute of Tropical Medicine, Antwerp, Belgium; 3 Telethon Kids Institute, University of Western Australia, Perth, Western Australia, Australia; 4 Université Pédagogique Nationale, Kinshasa, Democratic Republic of Congo; 5 University of Antwerp, Antwerp, Belgium; 6 Institut National de Recherche Biomédicale, Kinshasa, Democratic Republic of Congo; 7 Université de Kinshasa, Kinshasa, Democratic Republic of Congo; Common Heritage Foundation, NIGERIA

## Abstract

**Background:**

Socio-cultural and economic factors constitute real barriers for uptake of screening and treatment of Human African Trypanosomiasis (HAT) in the Democratic Republic of Congo (DRC). Better understanding and addressing these barriers may enhance the effectiveness of HAT control.

**Methods:**

We performed a qualitative study consisting of semi-structured interviews and focus group discussions in the Bandundu and Kasaï Oriental provinces, two provinces lagging behind in the HAT elimination effort. Our study population included current and former HAT patients, as well as healthcare providers and program managers of the national HAT control program. All interviews and discussions were voice recorded on a digital device and data were analysed with the ATLAS.ti software.

**Findings:**

Health workers and community members quoted a number of prohibitions that have to be respected for six months after HAT treatment: no work, no sexual intercourse, no hot food, not walking in the sun. Violating these restrictions is believed to cause serious, and sometimes deadly, complications. These strong prohibitions are well-known by the community and lead some people to avoid HAT screening campaigns, for fear of having to observe such taboos in case of diagnosis.

**Discussion:**

The restrictions originally aimed to mitigate the severe adverse effects of the melarsoprol regimen, but are not evidence-based and became obsolete with the new safer drugs. Correct health information regarding HAT treatment is essential. Health providers should address the perspective of the community in a constant dialogue to keep abreast of unintended transformations of meaning.

## Author Summary

The principal strategy for the control of HAT is based on early detection and prompt treatment of identified cases. A range of taboos are associated with HAT treatment in DRC. The origin of these taboos is not well understood. These taboos constitute major issues for patients and their families, lead to huge social pressure from the community on HAT patients and add in themselves to the burden caused by the disease itself. The aim of this study is to document the origin of these taboos and other cultural factors that are associated with HAT treatment, since an improved understanding of these factors and their implications may lead to strategies for improved community adherence to HAT screening and treatment. We found that the taboos are associated with the melarsoprol toxicity and have been established empirically following past interactions between healthcare providers and communities. The prohibitions started as simple instructions provided by healthcare providers about the management of HAT cases, but over time evolved into the community-based taboos we observe now. Use of less toxic treatment alternatives for HAT, dissemination of correct information regarding HAT treatment regimens, possible occurrence of adverse events and their cause would be beneficial to HAT control.

## Introduction

Human African Trypanosomiasis (HAT), also known as African sleeping sickness, is a neglected tropical disease that affects mainly poor populations living in rural areas [1–3]. HAT evolves in two clinical stages. The early haemo-lymphatic stage is characterized by non-specific clinical signs [4]. At this stage the infected persons do not yet feel the need to consult a health provider [4]. Those who feel sick usually have already progressed to the advanced (meningo-encephalitic) stage, which is characterized by an impairment of the central nervous system resulting in various neurological and psychiatric manifestations [4,5]. HAT treatment options are limited [6,7]. Early stage cases are given pentamidine, while the advanced stage of the disease was until recently treated with melarsoprol, an arsenic derivative that is known under its brand name “Arsobal” in HAT endemic areas. This late stage regimen is quite toxic and can cause fatal encephalopathy in five to ten percent of treated cases, in addition to other serious adverse events [4]. In recent years a combination treatment regime, NECT (Nifurtimox-Eflornithine Combination Therapy), has replaced melarsoprol as first-line treatment for second-stage HAT [4,8,9]. The NECT regimen is less toxic and causes fewer adverse events than melarsoprol [4]. However, logistical and financial factors may interfere with its deployment [10]. In the Democratic Republic of Congo (DRC), the supply of NECT to some of the country’s most remote endemic areas is challenging. Therefore melarsoprol is still being used instead of NECT in some areas. Melarsoprol is also still in use for refractory cases that have relapsed after NECT treatment. Simarro *et al*. (2012) reported that 12 percent of stage-2 HAT cases notified to the WHO were still treated with melarsoprol in 2010 [10].

Qualitative research conducted in the Democratic Republic of Congo (DRC) by Robays *et al*.*(2007)* in the province of Bandundu and by Mpanya *et al*.*(2012)* in the province of Kasai Oriental, identified several reasons why the population refrained from participating in HAT screening and treatment campaigns [11,12]. Fear of drug toxicity, financial considerations and the burden imposed by a series of “*taboos*” associated with the treatment regimen were the most important barriers. These *taboos* included prohibitions related to food (mainly around restrictions on the eating of sour and “hot” foods, both heated or spicy) and activities (do not engage in heavy work, do not walk in the sun, abstain from sexual intercourse). The community was strongly aware of these restrictions and said they were to be observed by HAT patients during treatment and for the following six months [12]. The nature of these restrictions and their duration negatively affected the participation rate of these communities in HAT screening as well as the treatment adherence of confirmed patients. It was unclear at the time where these taboos had originated from, whether they were self or health provider imposed, and what their rational basis was. Given their important impact on control efforts, we set out to document these perceptions and attitudes more in depth. Robays *et al*. (2007) in Bandundu province and Mpanya *et al* (2012) in East Kasai province reported prohibitions or taboos after treatment as barriers for uptake of the HAT control strategies [11,12]. In this study, we specifically investigated the origin of these taboos and explore how the HAT control program should address those barriers. A better understanding of these beliefs and their origin can lead to informed strategies for the DRC’s HAT control program to address these barriers and further improve the coverage of ongoing screening and treatment activities.

## Methods

### Background

HAT control is based on early detection and treatment of patients and vector control. Control programs conduct active screening campaigns in the villages at risk. Health facilities will also detect cases when people seek relief for their symptoms at their own initiative, which is known as passive screening. The diagnostic process is a three-step procedure. First, an antibody detection test is used as screening test (the Card Agglutination test for trypanosomiasis (CATT) or a HAT rapid diagnostic test). All positives are considered as suspects and subjected to parasitological confirmation before staging by lumbar puncture and treatment. During a two year period after treatment, all patients are invited every six months for a follow up which includes clinical and parasitological examinations as well as a lumbar puncture. Currently, systematic follow-up after treatment for HAT is no longer be recommended by WHO [4].

The non-participation of the community in HAT screening and treatment activities significantly compromises the ability of the national control program to attain its objectives [13]. The lack of community participation can be explained by multiple factors, including a number of community-enforced prohibitions that have been associated with the treatment of HAT [11,12]. An improved understanding of these prohibitions, their origin and their rationale is required in order to improve HAT control strategies in DRC as coordinated by the Programme National de lute contre la Trypanosomiase Humaine Africaine (PNLTHA).

### Design

**Fig 1:**
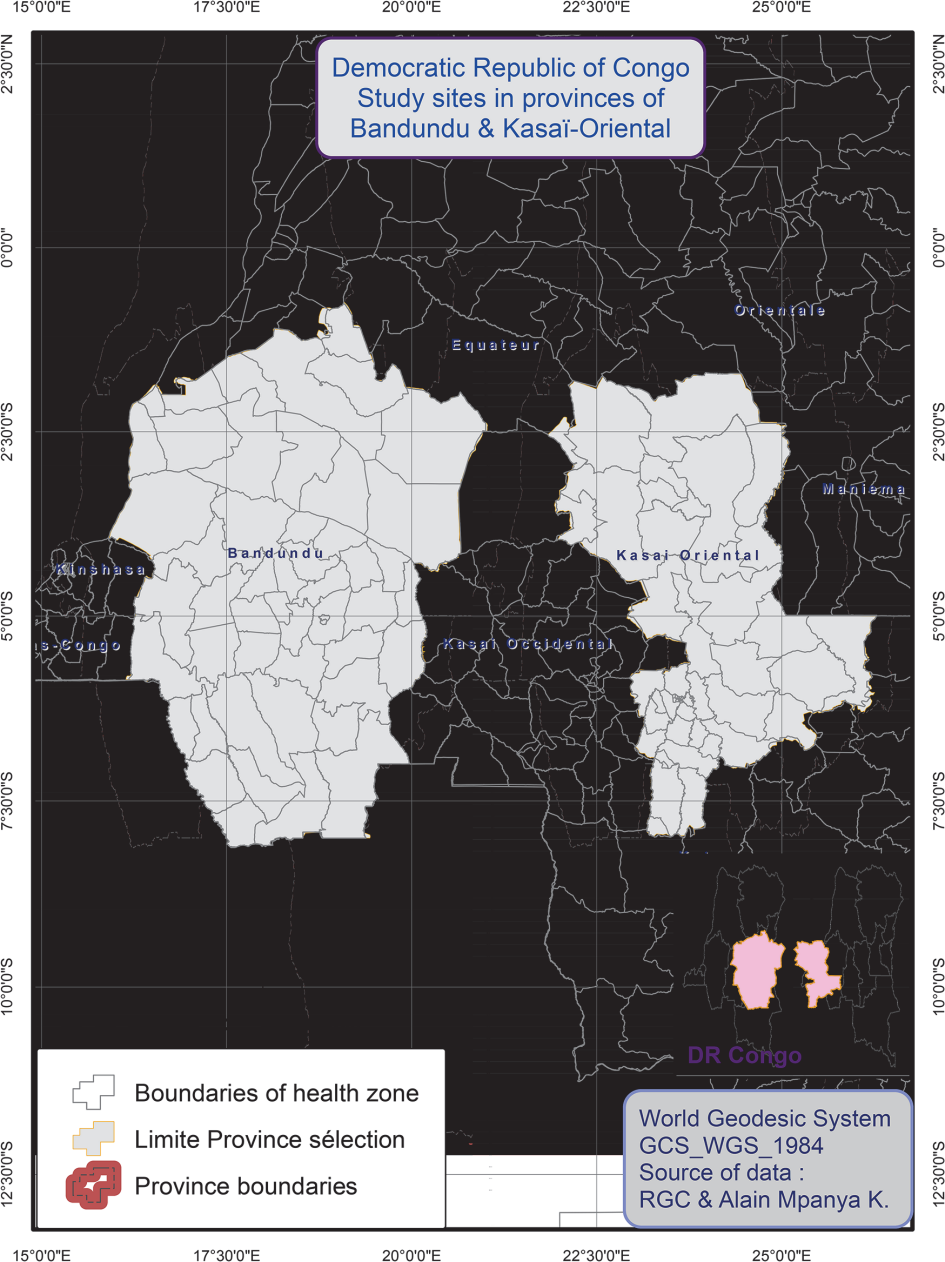
Location of the study sites.

We conducted a qualitative study combining semi-structured interviews and focus group discussions (FGD) to understand the origin and rationale of the beliefs towards HAT treatment and post-treatment follow-up in two provinces of the DRC: Kasaï Oriental and Bandundu (Fig 1). These two areas were chosen because they are highly endemic for HAT, and lagging behind in the control efforts as described by Hasker *et al*. in 2012 [14]. In order to investigate and document the origin of, and problems related to, these taboos, key personnel at all levels of the PNLTHA were interviewed. At the community level, we conducted FGDs with former HAT patients, followed by individual interviews with current HAT patients. Next, data were collected from the staff of dedicated treatment centres at the peripheral level, as well as the staff of the mobile units who are in charge of active screening of HAT patients in communities. Finally, at the central level we interviewed provincial and national coordinators of the PNLTHA. This bottom-up approach was used to identify the most knowledgeable interviewees.

The main themes in our question guides were: knowledge and perceptions regarding HAT treatment, advice that was given to the patients during treatment, after treatment and at follow-up, and finally the origin of the prohibitions that are associated with HAT treatment.

The interview/focus group discussion question guides were designed in French and then translated to the local languages of relevance to our study area (Kikongo or Tshiluba respectively for the Bandundu and East Kasai provinces). The question guides were developed by the investigators and were based on the outcomes of two previous studies [11,12] that identified the prohibitions associated with HAT treatment and their relevance to control initiatives in the region. The translations were verified by performing a reverse-translation of the question guides back into French by a second translator. The question guides were pre-tested in Kinshasa (in French) and non-study sites in Bandundu and East Kasai (in Kikongo and Tshiluba respectively). Transcripts were transcribed verbatim in the language of the interview or focus group discussion and where required translated into French for data analysis. These translations were verified by having a second translator perform a reverse translation of the transcripts back into the language of the interview or focus group discussion. The translation into English of the quotes used in this paper was verified amongst the co-authors.

### Focus group discussions

In total, eight FGDs were held with former HAT patients, six in Kasaï Oriental and two in Bandundu. We started our FGDs in Kasaï Oriental, following on with additional FGDs in Bandundu until we were confident a point of data saturation had been reached and no additional information was being captured. There were seven to eight participants in each FGD. Discussions with women and men were held separately to support free expression of opinion during the discussions. The FGDs were held in the local language and were facilitated by two co-investigators (a social anthropologist speaking Kikongo in Bandundu and a community health expert speaking Tshiluba in Kasaï Oriental). Both persons were well trained in these data collection methods. The principal investigator was present at all discussions and also helped to facilitate the FGD. The discussions were registered on a digital recorder and lasted on average 55 minutes per group.

From the community side of our study, focus group discussion participants were recruited among former HAT patients. Participants were identified based on a patient list provided by the national control program on the one hand, and with the help of health officers including community workers on the other. Permission was obtained from local authorities prior to inviting any former HAT patients to participate in the study. Potential participants were approached directly by the principle investigator or one of the community workers. All invited participants were provided with information about the aim of the study and informed consent was sought orally and recorded, after which the FGD was immediately started. The focus group discussions were performed in various places throughout the community where the participants felt comfortable to share their experiences. This varied from outdoors locations in the communities, to a classroom or a separate backroom in a church. The participants were offered a free soft drink after the discussion sessions, but no other incentives were provided (Table B in S1 Text).

### Semi-structured interviews

Twenty-four interviews were done with current HAT patients, nurses administering treatment, heads of mobile units and key personnel in the coordination of the PNLTHA. The language used during the interviews was the language preferred by the participant (Tshiluba, Kikongo or French). These interviews were recorded on a digital recorder and the duration was on average 40 minutes.

For interviews with HAT patients currently undergoing treatment, we selected those who were lucid and capable of speaking coherently.

For the service provider side of our study, we recruited current personnel of the national HAT control program as participants in our study. We selected interview participants on the basis of their seniority and time spent working on HAT control activities in the area. Here also informed consent was sought orally and recorded, after which the interviews were performed (Table A in S1 Text).

### Characteristics of FGD and interviews participants

We interviewed several categories of participants in this study. On the program delivery side, we interviewed senior staff of the national HAT control program. The interviewees consisted of two physicians, the two most senior technicians working at the provincial level of the HAT control program, the eight most senior heads of the mobile teams in the study area and seven nurses that work in the region's HAT treatment centre. In regards to the community, we interviewed seven HAT patients that were being treated at the time and organized nine focus group discussions with a total of 70 former HAT patients. Separate group discussions were organized for men and women in order to enhance active participation in the FGDs (Table 1). FGD participants consisted mainly of farmers and fishermen in Bandundu on the one hand, and diamond miners and farmers in Kasai Oriental on the other.

**Table 1:** Number of focus groups and interviews for each category of respondents.

**Categories**	**Focus groups**	**Interviews**
Former HAT patients	8	0
HAT patients	0	5
Nurses administering treatment	0	7
Heads of Mobile team	0	8
Coordinator Provincial level	0	2
Coordinator National level	0	2
**Total**	**8**	**24**

### Data analysis

The interviews and FGD were transcribed verbatim, translated into French and saved in an MS Word file prior to importation into the ATLAS.ti software package for analysis. We then “coded” the text thematically, a systematic and iterative process for reducing all the available transcript data into meaningful segments of text (or codes) [15]. These text segments were then labeled and organized into a coding tree, which provides a visual representation of the data structure. Our overall coding strategy for this study was developed on the basis of the interview and FGD question guides. Initial codes were generated on the basis of the themes present in the interview and focus group question guides, while additional codes were added progressively during the coding process as new themes and sub-themes emerged from the data. The principal investigator performed the coding process himself, after which the results were discussed and reviewed by the co-authors prior to finalizing the analysis. In this manuscript we present various quotes throughout the results section. These include a reference to the precise interview or FGD from which they were taken (Section F in S1 Text).

### Ethical issues

The study protocol was approved by the Ethics Committee of the School of Public Health of the University of Kinshasa, with the approval number ESP/CE/023/11. Informed consent was provided orally and was recorded on a digital audio device. Anonymity was guaranteed. Oral consent was deemed more appropriate than written consent given the local oral culture, and the consideration that requiring written consent might introduce a selection bias into our study given the socio-cultural context of the community.

## Results

### Perceptions regarding HAT treatment

The medicines used for the treatment of HAT are well known in the community. Pentamidine, the first phase drug, is perceived as the least toxic. The community does not associate it with any restrictions. “*Pentamidine*, *there are no taboos…”* (INT22). However, although healthcare providers do not consider it essential for patients on pentamidine, in their opinion all HAT patients should respect the health restrictions independent of HAT treatment type, in order to avoid confusion and a sense of discrimination *“… to protect those who take Arsobal*, *everyone should respect the taboos*.*”*(INT22).

The population knows melarsoprol under its brand name, “Arsobal^”^. Arsobal is perceived as a drug that is very toxic to humans. Respondents state that an injection of Arsobal provokes a strong feeling of heat and tingling in the whole body. Therefore Arsobal has been nicknamed “Tshiodomine”, a strong insecticide that is used locally. *“But the medication that we took*, *Arsobal*, *it is too strong*, *from the moment that we were injected*, *you feel the heat in the mouth and you pass out”* (FGD3). Arsobal is perceived as a drug that can cause severe and sudden side effects in any patient. All communities living in this region at high risk for HAT are afraid of having to undergo treatment with Arsobal. *“… but this man … although he was in good shape [before treatment began]*, *he’s dead now you see*, *so this aspect frightens the people”* (INT26). When someone is diagnosed with HAT, that person gets anxious at the thought of having to undergo treatment, and fears death as a result of treatment. Health care providers share this concern for the wellbeing of patients on Arsobal, since they also worry about how the patients will react to the drug. The associated fear in terms of sudden and potentially lethal side effects is thus shared by patients, their families and the treating nurses. *“Ah*! *It was fear*, *Arsobal*, *the patient wonders; Will I get through alive*? *And the nurses hope that hopefully they won’t react badly*.*”* (INT28). The healthcare providers state that managing the possible severe side effects of Arsobal is very difficult. Treating nurses feel that they lack the expertise and the financial resources to be able to deal with such adverse reactions, should they occur. They also find the issue of adverse effects difficult to discuss given the lack of an evidence base or comprehensive explanation regarding such adverse effects and their purported association with the prohibitions. When an adverse reaction does occur resulting from an Arsobal injection, a sense of helplessness seems to prevail. *“Arsobal*, *that’s the drug that exposes the patient to unpredictable and hardly controllable side effects”* (INT26). For providers and patients, it is also the drug that is most directly associated with health restrictions, the most frequently cited being: no hard labour, no sexual intercourse, no walking in the sun, no hot food, no alcohol, no smoking, no coffee, no spices, and no sour foods such as lemon, pineapple or sorrel.

NECT is the currently recommended drug regimen for first line treatment of second stage HAT. The community simply refers to it as “the new drug” and perceives it to be less toxic to the human organism. Interestingly, the new NECT regimen is deemed less toxic because it is diluted into the perfusion liquid prior to administration. *“But the drug seems to be mild*, *or maybe it is because it is put in the perfusion”* (FGD3).

Our study participants indicated that the community considers the restrictions associated with Arsobal mostly not to be relevant for NECT. The recent shift from Arsobal to NECT is therefore perceived as a liberation.*“DFMO (difluoromethylornithine) has abolished all the taboos of Arsobal*, *we can walk in the sun*, *work*, *we can eat hot fufu*,*…”* (FGD4). Alimentary taboos are completely abolished, and a patient who feels well is allowed to perform any kind of physical activity, with the important exception of sexual intercourse. This is still considered to be prohibited during, and for six months after treatment. *“The sole taboo is to have no sexual intercourse*, *but other things*, *you can do them if you have the strength …”* (FGD3). Unfortunately, the restriction on sexual intercourse is a significant one and is reported to be something that patients struggle to adhere to. *“But the big taboo was to have no sex with men for a period of six months”* (FGD6).

### On the origin of taboos

The taboos associated with HAT treatment appear to have originated from various sources and interactions between healthcare providers on the one hand and the communities they service on the other. The prohibition of physical activity, such as hard labour, sexual intercourse and walking in the sun seems to have existed for a very long time and probably found its origin in advice given by health providers. The heads of the mobile teams said that they had taken these recommendations from a book published by Burke in 1976 *“… doctor Burke (sic) wrote two books*, *we refer to these books*, *in the African medical guide it is also written that alcohol is forbidden during treatment”* (INT26). Burke was a former director of the central office of the HAT control program in DRC and has published a small handbook on HAT disease management. In the chapter concerning treatment, Burke recommended some precautions that needed to be taken before, during and immediately after administering a treatment regime of Arsobal, such as avoiding the consumption of alcohol, avoiding smoking and fatigue [16]. Concerning the recommendation about sexual intercourse, it is reported that this prohibition is based on experience from the field, as it was observed on occasions that those patients who had sexual intercourse during the six month rest period after receiving treatment had the tendency to experience severe sequelae, including death. *“Sexual intercourse for example*, *it’s based on the experiences that the sanitary agents observed in the field*.*”*(INT26), *“We consider sexual intercourse as hard labour”* (INT23). Regarding the prohibition of hard labour, our interviewees referred to the Belgian colonial law which exempted all persons suffering from HAT from performing hard labour, such as building roads. *“It’s in the law book of the Belgian Congo*, *it’s written there”* (INT26).

The nutritional taboos such as the restrictions related to the eating of hot food, spices and sour food seem to have their origins in community beliefs regarding health. “*Such as [not being allowed to eat] sorrel*, *mangoes*, *it’s what I respected*. *It’s what the people who had already undergone treatment told us*. *It wasn’t the nurse*.” (FGD1). Community members associated Arsobal with the concept of “heat”, since Arsobal was considered to cause a sensation of strong hotness throughout the whole body. In the traditional warm/cold nosology, a “hot” disease should not be made worse by additional exposure to heat such as through eating hot foods or walking in the sun. The rationale for this being that it would make the injected drug more active in the organism and produce severe side effects. Therefore avoiding all hot items is considered an important precaution after Arsobal treatment. *“A hot meal is forbidden*, *because if he eats it*, *it will heat up the blood and there will be a reaction”* (INT20). *“Arsobal is a strong drug that lights the fire in the body of people*, *afterwards it heats up the nerves*, *they must therefore remain in a chilled place”* (FGD3). Healthcare providers on the contrary do not believe in these alimentary taboos put forward by the community, but they have not intervened or otherwise tried to change the community’s beliefs in order to avoid confusion and contradictions.

### Importance of prohibitions

The health restrictions associated with Arsobal are perceived as the only way to protect oneself against the severe side effects of HAT treatment. *“They have recommended these taboos to avoid death”* (FGD1). The community and healthcare providers alike believe in these taboos, though not to the same extent, as highlighted above. As a result of strict social control, the patients are forced to adhere to these restrictions during the whole period of treatment and the full six months post-treatment. Moreover, the community condemns a patient that violates these taboos. The whole community therefore supervises the patient to ensure they adhere to the restrictions. If the patient is found to violate the taboos, the community condemns them. Furthermore, if a severe side effect that is perceived to be associated with the HAT treatment does occur, then the patient is blamed and criticized for not adhering to the taboos *“I have seen many people that had the disease and violated the taboos*, *they became crazy and died”* (FGD4). What kills a HAT patient is not considered to be the toxicity of the drug in itself, but rather his or her non-adherence to the prohibitions. *“We say that it is (the lack of adhering to) these taboos that kill us*, *the heart is fragile”* (FGD6). Consequently, the treatment outcome of a HAT patient is perceived to be a function of the level of adherence to these taboos. *“If you adhere*, *you will get cured*, *but you will die if you don’t follow them”* (INT14).

For the community, not all taboos have the same weight in terms of how hard they are to respect. The most difficult taboo to adhere to is considered to be the one that restricts sexual intercourse from the start of the treatment regime until six months after. *“The recommendation that was difficult for me*, *was to have no sex with my wife*, *it was very hard”* (FGD4). Sexual intercourse was not only the most difficult taboo to adhere to, it was also the restriction that was considered to most likely result in death if it was ignored, since over time the community has observed that those who did not respect this rule have died *“of all recommendations*, *sometimes you can accidently violate it and you will not die*. *However*, *concerning sexual intercourse with the wife*, *in reality many people died”* (FGD6). Given this belief, the sexual intercourse taboo is considered hard to deal with, since the patient lives in constant fear of dying. In combination with the constant surveillance of the family and wider community, this causes severe stress to the patient. *“I had to follow the recommendations because what I saw from others makes me afraid*. *You are seated here and someone goes sleeping with his wife*. *A while later he starts to sweat and dies in the room”* (FGD4).

### Impact on households

After having undergone treatment for HAT, patients are systematically expected to abide to a resting period of six months, which includes enforced adherence to the restrictions described above. Only after a scheduled six month follow up health check by a nurse may they resume their normal activities if the test results are satisfactory *“It has to be respected up to six months*, *no hard labour*, *you aren't even allowed to pick up and move a heavy chair*. *You are expected to sit under a tree in the cool shadow*, *you should not walk long distances during this period of rest”* (INT24). In some cases, this initial period of rest is extended further *“My grand-mother said to me not to work and rest as long as the drug was working inside my body*, *I have rested the six months that were prescribed*, *and now myself*, *I have added three more months”*(FGD3). During this period of rest the patient is fully dependent on his or her family. If the patient is the head of the household and the main source of income, the family will struggle to survive. *“When the six month resting period is mentioned*, *if it’s a man*, *he would respond; If I am not able to work for six months*, *how will we live*? *How will I support the family*? *How*? *In the end*, *my wife will leave me…”* (INT22).

An additional stress on the household ensues from the prohibition of sexual intercourse. In order to avoid temptation, the spouse undergoing HAT treatment is often obliged to spend time away from the household during this period. This can impact family life in many ways and is even considered to constitute a risk for divorce *“For the resting period*, *it torments me a lot*, *it provokes hunger*, *the household risks falling apart*, *men and women can even divorce…”*(FGD5). In addition, the patient is not allowed to take other drugs during their period of rest, even when they are diagnosed with another disease. *“I could only take paracetamol and no other medication*, *I followed these rules and I felt good afterwards”* (FGD3).

Last but not least, HAT patients carry a clear stigma, as they are thought to suffer from a bout of madness. They are considered incapable of rational thought. This kind of discrimination was reported to be particularly common in communities where only a limited number of persons were affected by the disease. However, when the number of patients rises in the community, this form of discrimination decreases as the disease becomes more “mainstream”*“me*, *in the beginning I considered the disease disgraceful*, *because if they know that you have it*, *the whole village will start talking*, *so we are embarrassed*, *people gossip and laugh a lot about it …”* (FGD4).

### Lumbar puncture at medical check-ups

The first follow-up health check at six months after finalising treatment generally puts an end to the resting period. This medical check consists of a lumbar puncture to assess cure, a procedure towards which the community also expressed trepidation. This anxiety was not only due to the procedure itself, but is also brought on given the implications in terms of not being able to work and perform agricultural activities after having undergone the test. This fear also affects people’s decision to show up for the mobile team’s screening activities in the first place. *“Each time that we have to undergo this test procedure*, *the injection that is done in our back can cause pain and result in a period of inactivity for one or two months*, *during which the agricultural work suffers*.*”* (FGD4). *“Here*, *especially in Kwango*, *the people didn’t want treatment*, *they were afraid of the lumbar puncture*, *they said that if you have a lumbar puncture done*, *you will never give birth again”* (INT22).

## Discussion

This study has documented various community beliefs and perceptions that are associated with the treatment of HAT in two provinces of DRC, and that are to a large extent shared by health care providers. Melarsoprol is well known and is perceived to be highly toxic to the human being. It is nicknamed “the taboo drug” as it is associated with a series of prohibitions that HAT patients are expected to adhere to. NECT is recognised as a new, less toxic drug that has resulted in the abolition of most of the taboos previously associated with melarsoprol, with the important exception of the ban on sexual intercourse. These restrictions emerged from the interactions between care providers and the communities they serviced, and most were originally intended to reduce the incidence of adverse drug effects caused by melarsoprol, without any firm evidence base. The prohibitions originating from the health care providers were amplified at community level and merged with traditional nosological interpretations of a symbolic nature. Communities consider the occurrence of any adverse events during and after treatment to be due to the patient’s non-adherence to the taboos. As a result, there are strong social control mechanisms in place in the communities to ensure that the patient observes the restrictions, a practice which in itself becomes a burden to the patient. Furthermore, the fear of the restrictions becomes in itself a deterrent to participate in HAT screening and treatment. Some community members argue it is better not to be tested (especially if they do not feel unwell) than to be ‘caught’ with the disease and to have to face the possible fatal outcomes of treatment. Remarkably, even the melarsoprol related iatrogenic deaths are attributed to the non-observance of taboos, not the toxicity of the drug per se.

We acknowledge that this study has some limitations. It was conducted in the two provinces in DRC with the highest HAT prevalence, where the existence of HAT treatment related taboos had already previously been identified [11,12]. We have not explored these aspects yet in provinces where HAT prevalences are lower. Failure rates and adverse event rates of melarsoprol do vary across regions, with e.g. 19.5% failures in the northern Equateur, 0.45% in the Bandundu and 29.6% in the Kasai Oriental [17,18]. Therefore community perceptions may vary geographically, as a result of epidemiological but also cultural heterogeneity. Another limitation of our study was that most of the study participants were patients who were in the second stage of the disease and were manifesting neuropsychiatric problems, which might have affected the nature of their responses. Nevertheless, by and large, our findings are congruent with those of other socio-anthropological researchers in the field of HAT and other tropical diseases.

One of the main findings of this study is the traditional nosological categorisation of illness into symbolic hot/cold categories by the community. HAT patients describe a sensation of heat going through their body when they are injected with melarsoprol. The drug is therefore considered to be a strong, mostly “hot” treatment leading to potentially serious side effects. This notion is directly related to the ban on further exposure to heat (no walking in the sun) or hot foods (no warm meals and hot spices) [12]. Similar mechanisms have been described previously and are framed within traditional approaches to health and disease. The idea of returning balance to the body when being affected by a hot or a cold disease by eating cold or hot food respectively has also been documented in Asian communities for diseases such as measles, smallpox and chickenpox [19–21].

The post therapeutic rest period of six months that is systematically prescribed to HAT patients is perceived as a significant problem: (i) the interdiction of sexual intercourse is considered as the most difficult to adhere to, since it may entail serious social consequences and may disrupt family life because of the forced abstinence [12]; (ii) it implies a loss of income due to an inability to work. This can come at a significant expense to the family. This issue was also noted by Lutumba *et al* (2007) in a rural community of Kinshasa, where the mandatory resting period after HAT treatment led to a significant loss of income [22]. The fear of this economic loss became in itself a barrier to participation in HAT screening activities. Mpanya (2012) and Robays (2007) came to a similar conclusion in their work in Kasaï Oriental and Bandundu where the fear of no longer being able to engage in subsistence agriculture was identified as a reason for which community members refused to be screened or treated for HAT [11,12].

The toxicity of melarsoprol is well established and is an issue that has been reported by various authors [7,10,23–25]. The adverse events of melarsoprol can be very severe [4,7]. Convulsions and other neurological disorders can precede coma and death during reactive encephalopathy in 5–10% of treated patients [7]. Simultaneous administration of steroids and thiamine was tried to improve the tolerance of the melarsoprol [26,27]. NECT can sometimes cause serious adverse events such as seizures and encephalopathy, but at a much lower frequency [28,29].The communities at risk knew the melarsoprol related adverse events including death very well [11,12]. As the mechanism of these severe adverse events (SAE) is unknown [4,30], and as care providers were not able to provide a rational explanation for them, this lack of information could have contributed to the emergence of a number of taboos that accompany HAT treatment.

Gouteux and Malonga (1985) noted in a socio-entomological household survey performed in a HAT focus in Yamba, Congo Brazzaville that treatment with melarsoprol was perceived to be excessively painful and dangerous [31]. Study participants stated that this was the reason why they did not seek care. The treatment of stage 2 HAT with melarsoprol is experienced by the patient, their family and care providers as a time of constant uncertainty and fear, since all involved are never sure whether the patient will successfully finish the treatment without a serious adverse reaction occurring. The treatment is perceived as more dangerous to the patient than the disease itself. Moreover, given the increasing number of treatment failures that have been reported in recent times with melarsoprol, a HAT patient may have to undergo the same treatment several times. Melarsoprol resistance rates up to 30% have been reported in several countries, such as Angola, Sudan, Uganda and DRC [4]. This issue of treatment failure and repeated treatment regimens may explain some of the social psychosis that exists around HAT treatment and the development of these taboos [12].

Uranw *et al* (2013) also noted that treatment failures and a lack of knowledge regarding adverse events were among the main factors for non-adherence to treatment for visceral leishmaniasis [32]. In their analysis of the barriers associated with access to treatment for Chagas disease, Manne (2013) noted that a lack of understanding of the disease and treatment (including adverse events) on behalf of health care providers and community members was a major barrier to treatment adherence [33].

Melarsoprol is no longer recommended as the first-line treatment for second stage *T*.*b*. *gambiense* HAT. It does however continue to be used in the case of NECT treatment failure, and also to a limited extent in some areas where NECT coverage remains sub-optimal due to technical and logistical reasons. Melarsoprol remains still in use as treatment for second stage *T*.*b*. *rhodesiense* HAT [4].

In conclusion, medical advice related to the management of HAT has transformed into a series of strictly enforced taboos that are entertained by care providers and community members in these regions of DRC. They are an important determinant of sub-optimal uptake of HAT screening campaigns. These taboos might well be abandoned if safer and more effective treatment options are made available more widely, as is already observed with the advent of NECT. This study showed once again the importance of correct, repeated and wide spread health information that is not disseminated in a one way fashion, but engages in a dialogue with the community. The challenge will be to establish a true communication with the community, which is very knowledgeable about the disease and the available therapeutic options. Health care providers should therefore avoid treating the community as though they are ignorant about the disease, since this clearly is not the case. It must be feasible to engage in a more culturally sensitive dialogue with the community, one which is in pace with the great therapeutic breakthroughs of recent years and translates evidence based health information in relevant terms. Providing health care providers and community members in HAT endemic areas with better information about treatment related effects might also prove beneficial for increasing the uptake of HAT control efforts.

## Supporting Information

S1 Text Table A: Characteristics of interviews. Table B: Characteristics of focus group discussions. Section A: Focus group discussion question guide for former patients. Section B: Interview guide for HAT patients. Section C: Interview guide for nurses administering treatment. Section D: Interview guide for Mobile team. Section E: Interview guide for national and provincial coordination. Section F: Coding structure.
(DOCX)

S1 Database Database taboos. (ZIP)
