# Supplementary material for: From Health Advice to Taboo: Community Perspectives on the Treatment of Sleeping Sickness in the Democratic Republic of Congo, a Qualitative Study
Source: PLoS Negl Trop Dis. 2015 Apr 9;9(4):e0003686. doi: 10.1371/journal.pntd.0003686 (PMC4391751; doi:10.1371/journal.pntd.0003686)
Supplement: S1 Text — Table B: Characteristics of focus group discussions. Section A: Focus group discussion question guide for former patients. Section B: Interview guide for HAT patients. Section C: Interview guide for nurses administering treatment. Section D: Interview guide for Mobile team. Section E: Interview guide for national and provincial coordination. Section F: Coding structure. (DOCX) [file pntd.0003686.s001.docx]

**S1 Text: Supporting information**

**Table A:** Characteristics of interviews

| **Interv nr** | **Category** | **Sex** | **Health facility type** |
| --- | --- | --- | --- |
| INT 7 | Nurses administering treatment | Female | Treatment center |
| INT 8 | Nurses administering treatment | Male | Treatment center |
| INT 9 | HAT Patient | Female | Treatment center |
| INT 10 | Nurses administering treatment | Male | Treatment center |
| INT 11 | HAT Patient | Female | Treatment center |
| INT 12 | Nurses administering treatment | Male | Treatment center |
| INT 13 | Nurses administering treatment | Male | Treatment center |
| INT 14 | HAT Patient | Male | Treatment center |
| INT 15 | HAT Patient | Female | Treatment center |
| INT 16 | HAT Patient | Female | Treatment center |
| INT 17 | Heads of Mobile team | Male | Mobile team |
| INT 18 | Heads of Mobile team | Male | Mobile team |
| INT 19 | Heads of Mobile team | Male | Mobile team |
| INT 20 | Heads of Mobile team | Male | Mobile team |
| INT 21 | Coordinator Provincial level | Male | Provincial coordination |
| INT 22 | Heads of Mobile team | Male | Mobile team |
| INT 23 | Heads of Mobile team | Male | Mobile team |
| INT 24 | Nurses administering treatment | Male | Treatment center |
| INT 25 | Nurses administering treatment | Male | Treatment center |
| INT 26 | Coordinator National level | Male | National coordination |
| INT 27 | Heads of Mobile team | Male | Mobile team |
| INT 28 | Coordinator Provincial level | Male | Provincial coordination |
| INT 29 | Coordinator National level | Female | National coordination |
| INT 30 | Heads of Mobile team | Male | Mobile team |

**Table B:** Characteristics of focus group discussions

| **FGD nr** | **Sex** | **Health zone** | **Province** | **Number of former patients** |
| --- | --- | --- | --- | --- |
| FGD 1 | Female | Bibanga | Kasai oriental | 8 |
| FGD 2 | Female | Bibanga | Kasai oriental | 8 |
| FGD 3 | Female | Kasansa | Kasai oriental | 8 |
| FGD 4 | Male | Bibanga | Kasai oriental | 8 |
| FGD 5 | Male | Kasansa | Kasai oriental | 8 |
| FGD 6 | Male | Bibanga | Kasai oriental | 8 |
| FGD 31 | Male | Masimaninba | Bandundu | 8 |
| FGD 32 | Female | Masimanimba | Bandundu | 7 |

**Section A: Question guide for focus group discussions for former patients**

1. **Knowledge on sleeping sickness:**

- What do you do when someone has sleeping sickness? Where do you take him/her?
- What do you think of the treatment against sleeping sickness?

1. **Taboos that accompany sleeping sickness:**

- What do you know about the advice given to the patients and their relatives during and after the treatment for sleeping sickness?
- Describe what a patient should do while he/she is on sleeping sickness treatment? (Probe towards list of taboos)
- Why do you think that patients should do those things?
- What will happen to the patient when he/she doesn’t follow the rules? (Probe towards individual, familial and community consequences)
- Who do you think provides advice about these aspects? (Probe towards the source)

1. **Scrutinize the taboos:**

- What do you think of people that say that during the treatment and resting period you should respect the following (probe towards origin and reason)
  - no hard labor
  - not walking in the sun
  - not eating hot meals
  - not drinking alcohol
  - no smoking
  - not eating hot peppers
  - no sexual intercourse

1. **Resting period of six months**

- What do you think of the six month resting period given to the patients after treatment?
- According to you, what is its importance?
- Where does this period come from? Who recommends it?

1. **Origin of the taboos linked to the treatment**

- According to you, where do all these recommendations during and after treatment come from? What is their origin?
- Since when have these taboos been introduced?
- The taboos were provided or introduced by whom?
- At which moment does the patient need to follow the taboos? (Probe towards period, duration)

1. **Expectations of former patients regarding the treatment of sleeping sickness**

- If you could recommend something for the improvement of management of sleeping sickness, what would it be?
- What would you suggest in regards to resolving the problems of the taboos that are linked to sleeping sickness treatment?
- Do you think it is possible to lift some of the taboos around sleeping sickness? (How?)
- Do you have any more comments to add?

**Section B: Interview guide for HAT patients**

1. **Knowledge on sleeping sickness:**

- What is your opinion on sleeping sickness?
- What is your opinion on the treatment of sleeping sickness that your are being given?

1. **Taboos linked to the treatment of sleeping sickness**

- What recommendations are given to HAT patients during and after treatment?
- Describe the things that sleeping sickness patients should do during treatment? (Probe towards list of taboos)
- Why do you think that the patients should follow these recommendations?
- What happens if a patient doesn’t follow these recommendations? (Probe towards individual, familial and community consequences)
- Who provides those recommendations? (Probe towards sources)

1. **Scrutinize the taboos**

- What do you think of people that say that during the treatment and resting period you should respect the following(probe towards origin and reason)
  - no hard labor
  - not walking in the sun
  - not eating hot meals
  - not drinking alcohol
  - no smoking
  - not eating hot peppers
  - no sexual intercourse

1. **Resting period of six months?**

- What is your opinion on the 6 months resting period after treatment?
- According to you, what is its importance?
- Where does this period come from? Who recommends it?

1. **Origin of the taboos linked to the treatment**

- According to you, where do all these recommendations -during and after treatment- come from? What is their origin?
- Since when have these taboos been introduced?
- The taboos were provided or introduced by whom?
- At which moment does the patient need to follow the taboos? (Probe towards period, duration)

1. **Expectation of the patients regarding the treatment of sleeping sickness**

- If you could recommend something for the improvement of management of sleeping sickness, what would it be?
- What would you suggest to resolve the problems regarding the taboos that have been linked to sleeping sickness treatment?
- Do you think it is possible to lift some taboos around sleeping sickness? (how?)
- Do you have more comments to add?

1. **Add questions on new relevant elements reported during the FocusGroup Discussions.**

…………………………………………………………………………………………………………………………………………………………………………………………………………………………

- Do you have more comments to add?

**Section C: Interview guide for nurses administering treatment**

1. **Knowledge on sleeping sickness**

- What do you think about sleeping sickness?
- What do you think about the treatment of sleeping sickness?

1. **Recommendations given to the patients and their relatives regarding the treatment of sleeping sickness**

- What are the recommendations given to the patients and their relatives during and after receiving treatment for sleeping sickness?
- Why do you give these recommendations to the patients?
- What happens if the patients don’t follow these recommendations?
- Where do these recommendations originate from? (Probe for origin and source of the information)
- Are there things that the patient should absolutely adhere to during treatment? Which things? (Probe for list of taboos)

1. **Scrutinize the taboos:**

- What do you think of those that say that during the treatment and resting period you should respect the following(probe towards origin and reason)
  - no hard labor
  - not walking in the sun
  - not eating hot meals
  - not drinking alcohol
  - no smoking
  - not eating hot peppers
  - no sexual intercourse

1. **Resting period of six months?**

- What is your opinion on the 6 months resting period after treatment?
- According to you, what is its importance?
- Where does this period come from? Who recommends it? (Probe for origin and source of information)

**5. Origin of taboos linked to the treatment**

- According to you, where do all these recommendations about what to do during and after treatment come from? (Probe for origin and information source)
- Since when have these taboos been around?
- The taboos were provided by whom?
- At which moment does the patient need to follow the taboos? (Probe towards period, duration)
- Is it an instruction given by the national HAT control program?

**6. Perspective towards sleeping sickness treatment**

- If you would be responsible of the national HAT control program, what would you do to improve the management of HAT patients?
- If you would be the director of the national HAT program, what would you do to resolve the problem concerning the taboos linked to the treatment?
- Do you think it would be possible to remove the taboos around sleeping sickness? (How?)

1. **Add questions on new relevant elements reported during the Focus Group Discussions.**

…………………………………………………………………………………………………………………………………………………………………………………………………………………………

- Do you have more comments to add?

**Section D: Interview guide for mobile teams**

1. **Knowledge on sleeping sickness**

- What is your opinion on sleeping sickness?
- What is your opinion on the treatment of sleeping sickness?

1. **Advice given to the patients and their relatives regarding the treatment of sleeping sickness**

- Are there any instructions that the patients and their relatives should follow during the treatment period for sleeping sickness? And after treatment? (Which ones?)
- Do you think that these instructions are important for the patients? (Explain?)
- What happens if the patients don’t follow the instructions?
- What is the origin of these instructions given to the HAT patients? (Probe for place/institution that teaches the instructions, at school? Somewhere else?)
- Are there things that the patients should adhere to while they are on treatment? What are those things? (Probe for the list of taboos)

1. **Scrutinize the taboos**

- What do you think of people that say that during treatment and resting period you should respect the following(probe towards origin and reason)
  - no hard labor
  - not walking in the sun
  - not eating hot meals
  - not drinking alcohol
  - no smoking
  - not eating hot peppers
  - no sexual intercourse

1. **Resting period of six months**

- After how much time does a HAT patient restart his/her daily activities?
- What do you think about the six-month resting period imposed on HAT patients?
- Do you think that this resting period of six months is important?
- Where does this rule come from? Who provided these instructions? (Probe for origin and source of information)

1. **Origin of the taboos linked to treatment**

- Do you know of the existence of instructions that HAT patients should follow during and/or after treatment? (Which ones?)
- According to you, where do these instructions come from? (Probe for origin and sources of information)
- When did these taboos appear?
- Are these taboos written down in a book? (Insist on provenance)
- Did the national HAT control program provide instructions regarding the management of HAT patients? (Which ones?)

1. **Perspective on treatment for sleeping sickness**

- Do you think that HAT patients receive proper treatment?
- What do you suggest to improve the management of HAT patients at the level of the treatment centre?
- What would you improve if you were responsible for the national HAT control program?
- Do you think it is possible to lift the taboos linked to the treatment of HAT? (How?)

1. **Add questions on new relevant elements reported during the FocusGroup Discussions.**

…………………………………………………………………………………………………………………………………………………………………………………………………………………………

- Do you have more comments to add?

**Section E: Interview guide for national and provincial coordination**

1. **Situation of HAT in DRC**

- What is your opinion on the importance/spread of HAT in DRC?
- Are there sufficient means to control HAT?

1. **Conceptions on HAT treatment**

- What do you think about HAT treatment?
- What is your opinion on the compliance of patients to HAT treatment?
- What problems exist regarding HAT treatment?

1. **Organization of HAT treatment**

- Are there recommendations that nurses should provide to the patients and their relatives in the period during and after HAT treatment? What are those recommendations?
- Why do the nurses have to give these recommendations to the patients?
- What happens to the patients when they don’t follow these recommendations?
- What is the origin of these recommendations that the nurses give to the patients and their relatives? (Probe for origin and source of the information)
- Are there instructions provided by the national HAT control program regarding these recommendations? (Probe for origin and source of the information)

1. **Scrutinize the taboos**

- What do you think of people that say that during the treatment and resting period you should respect the following (probe towards origin and reason)
  - no hard labor
  - not walking in the sun
  - not eating hot meals
  - not drinking alcohol
  - no smoking
  - not eating hot peppers
  - no sexual intercourse

1. **Resting period of six months**

- What are your thoughts regarding the six month resting period recommended to the patients after treatment? What does the patient do during this period?
- According to you, what is the importance of this period?
- Where does this resting period come from? Who imposes it on the patients? (Probe for origin and source of information)

1. **Origin of taboos linked to treatment**

- What is the origin of these taboos linked to HAT treatment? (Seek the source of the information)
- Since when have these taboos appeared? And by who were they introduced?
- Is it a recommendation from the national HAT control program?
- What is the position of the national HAT control program regarding these taboos?

1. **Perspectives on HAT treatment**

- What would you suggest in order to improve management of HAT patients if you were responsible for the HAT control program?
- What would you do to resolve the problems regarding the taboos linked to HAT treatment if you were the director of the national HAT control program?
- Do you think it is possible to lift the taboos linked to HAT treatment? (How?)

1. **Add questions on new relevant elements reported during the FocusGroup Discussions.**

…………………………………………………………………………………………………………………………………………………………………………………………………………………………

- Do you have more comments to add?

**Section F: Coding structure**

1. **Trait_THA: HAT treatment**
   1. Trait_Comm: Knowledge on HAT medication by the community (Current and former HAT-patients)
   2. Trait_percept_Comm: Perception of HAT treatment by the community (Current and former HAT-patients)
   3. Trait_Effect_Comm: Perceived Side effects of the treatment
      1. Trait_Effet_Comm: Side effects of the treatment perceived by the community
      2. Trait_Effet_Prest: Side effects of the treatment perceived by health care providers
   4. Trait_View: Opinion on HAT treatment
      1. Comm_Trait_View: Opinion of the community on HAT treatment
      2. Prest_Trait_View: Opinion of health care providers on HAT treatment
   5. Mal_Trait_Fait: What the HAT patient needs to do during treatment
   6. Inst_Trait: Instructions given to the patients before HAT treatment
   7. Rec_Trait: Recommendations on HAT treatment
2. **Inter_Trait: Taboos related to HAT treatment**
   1. Orig_Inter: Origin of Taboos related to HAT treatment
      1. Orig_Inter_Comm: Origin of Taboos related to HAT treatment coming from the community
      2. Orig_Inter_Prest: Origin of Taboos related to HAT treatment coming from health care providers
   2. Inter_View: Opinion on Taboos related to HAT treatment
      1. Comm_Inter_View: Opinion of the community on Taboos related to HAT treatment
      2. Prest_Inter_View: Opinion of health care providers on Taboos related to HAT treatment
      3. Prog_Inter_View: Opinion of the National HAT Control Program managers on Taboos related to HAT treatment.
   3. Comm_Inter_percept: Perception of the community on taboos
   4. Prest_Inter_Attit: Attitude of health care providers towards taboos
   5. Just_Inter: Reasons that justify the taboos
   6. Inter_Attit_Mal: Attitude of the patients towards the taboos
   7. Inter_Dur: duration during which taboos needs to be respected
   8. Inter_import: importance of taboos
   9. Sug_Inter: Suggestions regarding the management of the taboos
3. **Poid_inter: weight of taboos**
   1. Poid_Inter_mal: weight of the taboos on the patients
   2. Cons_Inter: Consequences when taboos are not respected
   3. Impl_Inter_contr: Implications of the taboos regarding HAT control
   4. Impact_Inter_Menag: Impact of taboos on households
4. **Rep_Trait: post-therapeutic rest**
   1. Orig_Rep_Trait: Origin of post-therapeutic rest
   2. Just_Rep_View: Reasons for post-therapeutic rest
   3. Rep_View: Opinion on post-therapeutic rest
   4. Rep_Instr: Instructions on post-therapeutic rest
      1. Prog_Rep_Instr: instructions on post-therapeutic rest given by the national and provincial coordination of HAT
      2. Prest_Rep_Instr: Instructions on post-therapeutic rest given by health care providers
      3. Comm_Rep_Instr: Instructions on post-therapeutic rest given by the community
   5. Comm_Rep_Prese: What the post-therapeutic rest represents to the community
   6. Cons_Rep_Trait: Consequences when post-therapeutic rest is not respected
   7. Ponct_Lomb: lumbar puncture at medical check ups
      1. Ponct_Lomb_Percept: Community perception of lumbar puncture
      2. Ponct_Lomb_Cons: perceived consequences of lumbar puncture
